# Gadoxetate-enhanced dynamic contrast-enhanced MRI for evaluation of liver function and liver fibrosis in preclinical trials

**DOI:** 10.1186/s12880-019-0378-5

**Published:** 2019-11-15

**Authors:** Jimi Huh, Su Jung Ham, Young Chul Cho, Bumwoo Park, Bohyun Kim, Chul-Woong Woo, Yoonseok Choi, Dong-Cheol Woo, Kyung Won Kim

**Affiliations:** 1 Department of Radiology and Research Institute of Radiology, Asan Medical Center, University of Ulsan College of Medicine, 88 Olympic-ro 43-gil, 138-736, Songpa-gu, Seoul 05505 Korea; 2 Department of Radiology, Ajou University School of Medicine and Graduate School of Medicine, Ajou University Hospital, Yeongtong-gu, Suwon 16499 Korea; 3 Center for Bioimaging of New Drug Development, Asan Institute for Life Sciences, Asan Medical Center, Songpa-gu, Seoul 05505 Korea

**Keywords:** Gadoxetate, Dynamic contrast-enhanced, Magnetic resonance imaging, Liver fibrosis, Liver function

## Abstract

**Background:**

To facilitate translational drug development for liver fibrosis, preclinical trials need to be run in parallel with clinical research. Liver function estimation by gadoxetate-enhanced dynamic contrast-enhanced MRI (DCE-MRI) is being established in clinical research, but still rarely used in preclinical trials. We aimed to evaluate feasibility of DCE-MRI indices as translatable biomarkers in a liver fibrosis animal model.

**Methods:**

Liver fibrosis was induced in Sprague-Dawley rats by thioacetamide (200 mg, 150 mg, and saline for the high-dose, low-dose, and control groups, respectively). Subsequently, DCE-MRI was performed to measure: relative liver enhancement at 3-min (RLE-3), RLE-15, initial area-under-the-curve until 3-min (iAUC-3), iAUC-15, and maximum-enhancement (Emax). The correlation coefficients between these MRI indices and the histologic collagen area, indocyanine green retention at 15-min (ICG-R15), and shear wave elastography (SWE) were calculated. Diagnostic performance to diagnose liver fibrosis was also evaluated by receiver-operating-characteristic (ROC) analysis.

**Results:**

Animal model was successful in that the collagen area of the liver was the largest in the high-dose group, followed by the low-dose group and control group. The correlation between the DCE-MRI indices and collagen area was high for iAUC-15, Emax, iAUC-3, and RLE-3 but moderate for RLE-15 (r, − 0.81, − 0.81, − 0.78, − 0.80, and − 0.51, respectively). The DCE-MRI indices showed moderate correlation with ICG-R15: the highest for iAUC-15, followed by iAUC-3, RLE-3, Emax, and RLE-15 (r, − 0.65, − 0.63, − 0.62, − 0.58, and − 0.56, respectively). The correlation coefficients between DCE-MRI indices and SWE ranged from − 0.59 to − 0.28. The diagnostic accuracy of RLE-3, iAUC-3, iAUC-15, and Emax was 100% (AUROC 1.000), whereas those of RLE-15 and SWE were relatively low (AUROC 0.777, 0.848, respectively).

**Conclusion:**

Among the gadoxetate-enhanced DCE-MRI indices, iAUC-15 and iAUC-3 might be bidirectional translatable biomarkers between preclinical and clinical research for evaluating histopathologic liver fibrosis and physiologic liver functions in a non-invasive manner.

## Background

There have been great efforts to develop new drugs for liver fibrosis and chronic hepatitis in the past decades. Accordingly, the need to monitor the degree of liver fibrosis/function in a non-invasive and reproducible manner is increasingly important in preclinical trials as well as clinical research [1]. Recently, the concept of bidirectional translation with “from preclinical to clinical” and “from clinical to preclinical” has been emphasized to facilitate drug development in parallel manner in both sides.

Gadolinium ethoxybenzyl diethylenetriaminepentaacetic acid (Gd-EOB-DTPA; hereafter referred to as gadoxetate) is a dual-function contrast agent for magnetic resonance imaging (MRI). In the vascular phase, gadoxetate acts as an extracellular contrast agent that can be used to evaluate hemodynamic change or vascularity. In the later phase, gadoxetate acts as a hepatocyte-specific contrast agent to enhance the hepatocytes [2, 3]. The characteristics of gadoxetate as a hepatocyte-specific contrast agent allow the evaluation of liver function [4, 5].

Dynamic contrast-enhanced MRI (DCE-MRI) can characterize the functional aspects of biological tissues by using both the temporal information and spatial information provided by MRI [6, 7]. Liver function is generally assessed by indocyanine green (ICG) tests such as the ICG retention rate at 15 min (ICG-R15) or ICG clearance test. In the liver tissue, gadoxetate and ICG used the same receptors such as OATP and MRP; theoretically, DCE-MRI results would reflect those of the ICG test.

Shear wave elastography (SWE) is an ultrasonographic technique to measure liver stiffness. In clinical practice, the use of SWE has been rapidly increasing. Many vendors have incorporated the SWE into their ultrasonography machines [8]. Nowadays, SWE is used in both clinical trial and preclinical trials.

Gadoxetate-enhanced DCE-MRI is in the clinical validation stage, in which whether gadoxetate-enhanced DCE-MRI biomarkers can reflect the pathologic process and clinical outcomes and whether the method can produce results in a reproducible manner are evaluated [7]. The gadoxetate-enhanced DCE-MRI biomarkers used in clinical research and practice can be translated into the preclinical trials. However, gadoxetate-enhanced DCE-MRI is rarely used in preclinical trials due to a lack of validation and evidence [9]. Nevertheless, the use of gadoxetate-enhanced DCE-MRI in preclinical trials might be a powerful tool provided that the validity of biomarkers and technical feasibility are ensured. Therefore, we performed an animal study to evaluate biomarkers in a rat liver fibrosis model.

## Methods

### Animal model

All experiments associated with this study were approved by our institutional animal care and use committee (No. 2015–13-117). This study follows the ARRIVE Guidelines for reporting animal research [10].

All Sprague-Dawley (SD) rats (male, 8 weeks old, 270–280 g) were obtained from Orient Bio (Seoul, Korea) and maintained under specific pathogen-free conditions. Rats were monitored daily during experimental period for body weight, and general body condition, such as appearance, food/water intake, respiration and ambulation. Animals were euthanized when showing signs of distress and when the weight loss exceeding 20% of body weight. When sacrificing or euthanizing animals, we used carbon dioxide inhalation method using a dedicated euthanasia chamber for rodents.

A total of 24 SD rats were randomly assigned into three groups (high-dose, *n* = 8; low-dose, *n* = 8; control, *n* = 8). Liver fibrosis was induced in SD rats using thioacetamide (Sigma-Aldrich Co., St. Louis, MO, USA), which is a hepatotoxic agent that causes centrilobular necrosis and liver fibrosis [8]. Thioacetamide was administered intraperitoneally three times per week for 8 weeks with the following dose: 200 mg/kg for the high-dose group, 150 mg/kg for the low-dose group, and saline for the control group. The dose of thioacetamide was determined by a preliminary experiment using five doses (two rats per each dose) escalated from 0 mg/kg (vehicle only) to 100 mg/kg, 150 mg/kg, 200 mg/kg, and 250 mg/kg according to previously reported literatures [11–13]. In our preliminary experiment, the 100 mg/kg dose did not induce histologic liver fibrosis consistently, and the 250 mg/kg dose resulted in death during the thioacetamide medication period. Therefore, we decided to use 150 mg/kg and 200 mg/kg in this experiment.

### DCE-MRI acquisition

The MRI scan was performed after 8 weeks of thioacetamide administration. A 3-T MR scanner (Magnetom Skyra; Siemens Healthcare, Erlangen, Germany) with a 16-channel hand/wrist coil was used. The rats were anesthetized during the imaging session with 1.3–1.5% isoflurane/air mixture. DCE-MRI including T1 mapping was performed with CAIPIRINHA-VIBE, which has been established as a motion-insensitive and fast scanning method. CAIPIRINHA-VIBE was performed with the following parameters: TR/TE 4.3/1.5 ms, flip angle 25°, matrix size 128 × 128, and an acceleration factor of 4 (2 each in the phase- and partition-encoding directions) with a reordering shift of 1. The scan coverage of this sequence was 78 mm (52 slices × 1.5 mm thickness), and the field of view was 100 × 100 mm, which was sufficient for covering the entire liver in all the rats.

For DCE-MRI scanning, the T1 map was generated with the variable flip-angle technique (α = 2°, 8°, 15°, 22°, 29°) without contrast agent injection. During dynamic scanning, five-phase baseline acquisitions were performed before contrast agent injection. When the sixth phase acquisition was started, 0.05 mmol/kg body weight (0.015 mmol for rat with 300 mg body weight) of gadoxetic acid (Eovist or Primovist; Bayer Healthcare, Berlin, Germany) was manually administrated by hand injection as a bolus using an 0.5 mL insulin syringe with 31 gauge needle (BD Ultra-Fine II insulin syringe; Becton Dickinson and Company, Franklin Lakes, NJ). To prepare 0.015 mmol gadoxetic acid solutions, the commercial pre-filled syringe of 0.25 mmol/mL was diluted 5-fold using normal saline to generate 0.05 mmol/mL solution, then 0.3 mL was loaded in the insulin syringe. Then, a dynamic series was repeated every 3.6 s for 3 min followed by every 60 s for 30 min.

### DCE-MRI analysis

We developed a comprehensive software, Asan J, by combining Image J (NIH, Bethesda, MA, USA) and MATLAB (The MathWorks, Natick, MA, USA). Our software contains modules for evaluating liver function using the signal intensity (SI) measured by MRI.

The SI was measured on a pixel-by-pixel basis using the Asan J software. An experienced radiologist (J. H.) with more than 9 years of experience measured the SI of the rat liver at three different regions of interest (ROIs) in the liver parenchyma, avoiding enhancement of vessels and bile structures. Another experienced radiologist (K.W.K.) with more than 12 years of experience double-checked the ROIs. The mean SI of each ROI was recorded and used for analysis.

The time-signal intensity curve was reconstructed for each ROI. Based on the time-signal intensity curve, the values of the relative liver enhancement (RLE) at 3 min (RLE-3) and 15 min (RLE-15) after contrast agent injection were calculated using the following formula [7, 14]:

$$ \mathrm{RLE}=\left({\mathrm{SI}}_{\mathrm{Liver}-\mathrm{enh}}-{\mathrm{SI}}_{\mathrm{Liver}-\mathrm{unenh}}\right)/{\mathrm{SI}}_{\mathrm{Liver}-\mathrm{unenh}} $$

Based on the time-signal intensity curve, the values of the initial area of under the curve (iAUC) until 3 min (iAUC-3) and until 15 min (iAUC-15) after contrast agent injection were calculated by integration of the time-signal intensity curve [15]. The maximum enhancement (Emax) was also acquired.

### Shear wave Elastography

At the same day of DCE-MRI scanning, two-dimensional SWE measurements were also acquired with an Aplio 500 Platinum ultrasound machine (Toshiba Medical Systems Corporation, Tokyo, Japan) using a linear probe (PLT-1005BT transducer (7.0–14.0 MHz). After shaving the upper abdomen, liver stiffness measurements were taken from the left hepatic lobe. The operator measured the SWE using at the similar depth of 1 cm from the liver surface and recorded the liver stiffness in kilopascal (kPa).

### Histopathology

After MRI examination, the animals were euthanized, and the liver was excised. The excised tissues were fixed in 10% formalin, and paraffin blocks were made. For microscopic evaluation of the liver parenchyma, hematoxylin and eosin (H&E) staining was performed. To evaluate the extent of liver fibrosis, Masson’s trichrome staining was performed using a commercially available kit (Sigma-Aldrich, Seoul, Korea). In Masson’s trichrome staining, the cytoplasm and muscle fibers would be stained red, whereas the collagen would be stained blue [16, 17].

The collagen area was quantified using Image J software (NIH, Bethesda, MD, USA) with the following steps: (1) Three representative hotspots were determined at a lower magnification (40×); (2) Those areas were captured and digitized for morphometric analysis; (3) The collagen area was selected with the specified colorimetric threshold (blue color). If the collagen area was not selected automatically by Image J, we adjusted the area manually based on the H&E staining. The measurement values from the three hotspots were averaged and used for statistical analysis.

### ICG test

To evaluate liver function, ICG-R15 was determined, which is the most widely used method in clinical practice [18]. The ICG-R15 value is the ratio between the ICG concentration 15 min after injecting ICG (C-15) and the initial concentration (C-0), calculated by the formula: ICG-R15 (%) = C-15/C-0 × 100. The higher level of ICG-R15 test result means the lower level of liver function.

ICG (Daiichi Sankyo, Tokyo, Japan) was dissolved in normal saline to a final concentration of 2.5 mg/mL. The right carotid artery was surgically exposed and cannulated for blood sampling. The ICG solution with a concentration of 2.5 mg/kg body weight was injected through the tail vein. The blood sample was obtained at 15 min after ICG injection and mixed with 20 μL of ethylenediaminetetraacetic acid (EDTA). The blood sample was then centrifuged to obtain the plasma. The plasma sample was diluted in 1% bovine serum albumin solution. The C-15 was measured spectrophotometrically at 805 nm and the C-0 was calculated by estimating that there is 2.5 mg/kg ICG in a rat with a plasma volume of 40 mL/kg body weight [19], yielding 16 mg/mL.

### Statistical analysis

The quantitative indices (DCE-MRI indices, SWE, ICG-R15, and histologic collagen area) were compared between the three groups, i.e., control, low-dose, and high-dose group, by one-way analysis of variance (ANOVA) with post-hoc analysis using the Tukey-Kramer method.

The correlation between these quantitative indices was calculated using the Pearson correlation coefficient. The value of correlation coefficient is interpreted as follows: less than 0.30, negligible; 0.30–0.50, low; 0.50–0.70, moderate; 0.70–0.90, high; 0.90–1.00, very high [20].

The accuracy of diagnosing liver fibrosis was evaluated by receiver-operating-characteristic (ROC) curve analysis and area under the ROC (AUROC). A *p* value of < 0.05 was considered to indicate a statistically significant difference. MedCalc (version 17.7.2; MedCalc Software bvba, Ostend, Belgium) and SPSS Statistics for Windows (version 21.0; IBM Corp., Armonk, NY, USA) were used.

## Results

### Animal models

Among the 24 rats, 2 rats died during the 8 weeks of thioacetamide administration. Finally, 8 rats in the control group, 6 rats in the low-dose group, and 8 rats in the high-dose group were included in this study. Signs of toxicity, such as ruffled fur, anorexia, cachexia, skin tenting, skin ulcerations, or toxic death [21], were not observed in any of the rats that survived.

**Fig. 1:**
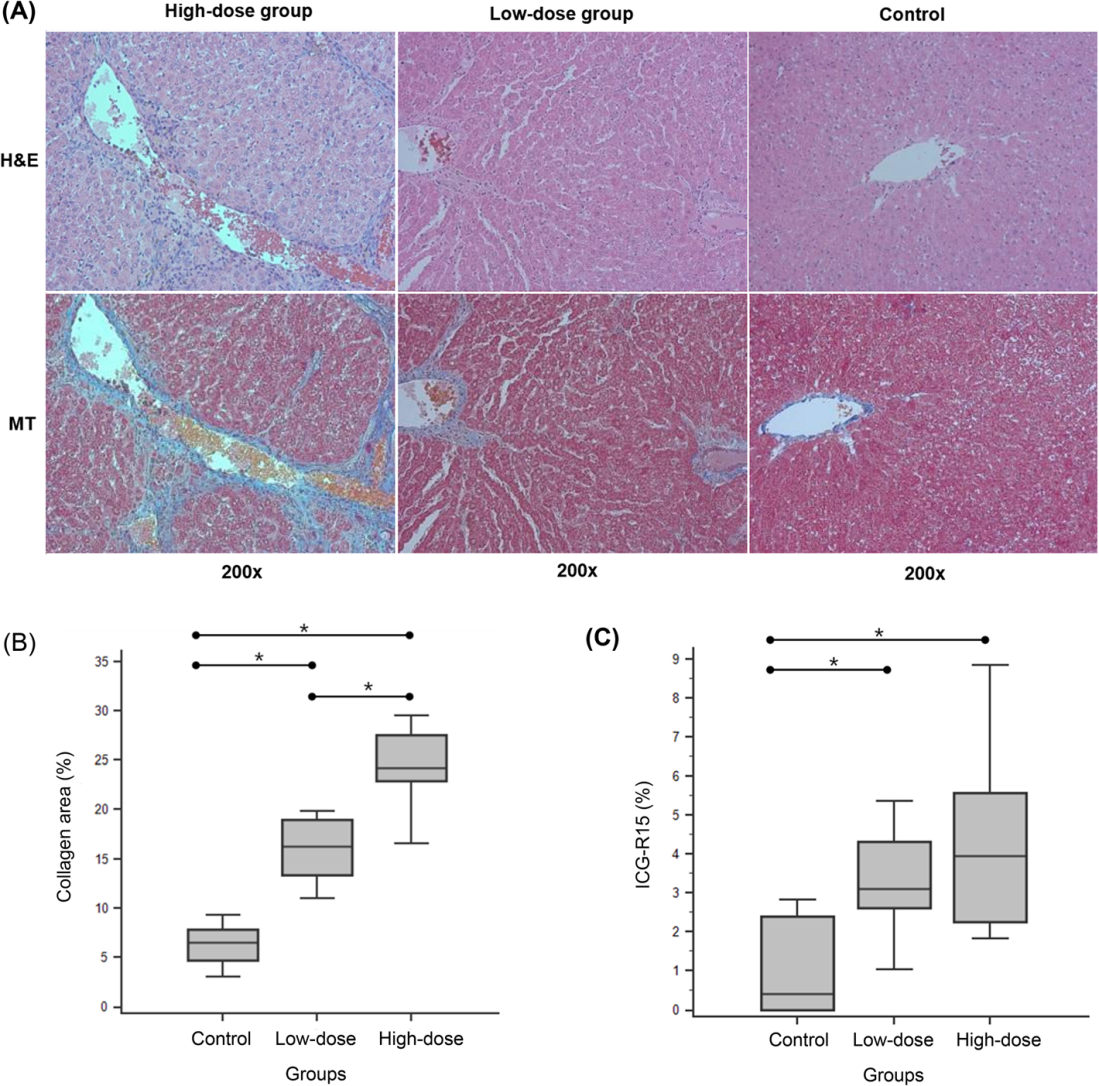
Histopathology of the liver parenchyma. **a** Hematoxylin and eosin (H&E) stain and Masson’s trichrome (MT) stain at 200× magnification. The H&E stain demonstrates centrilobular necrosis and scattered inflammation which are more severe in high-dose group than low-dose group. In the MT stain, the liver fibrosis is depicted as the fibrous bands or septa with abundant collagen deposition which is stained as blue color. **b** Comparison of the collagen area (%) between groups. Asterisks (*) refer the pairs with a statistically significant difference. **c** Comparison of the ICG-R15 (%) between groups. Asterisks (*) refer the pairs with a statistically significant difference

All rats in the control group showed normal histologic results without fibrosis, inflammation, and steatosis. In all rats in the low-dose group and high-dose group, H&E staining revealed damaged hepatic cells with apparent toxicity characterized by periportal hepatocyte vacuolation, centrilobular necrosis, heavy pigmentation around central veins, scattered inflammation, and giant cell transformation. The results of Masson’s trichrome staining indicated that liver fibrosis with abundant collagen deposition was successfully induced, and the fibrous bands or septa originate from the portal areas and extend into the hepatic parenchyma (Fig. 1a).

The collagen area (%) of the liver specimens was the largest in the high-dose group (24.9% ± 4.6), followed by the low-dose group (16.1% ± 3.3) and control group (6.3% ± 2.1), as shown in Fig. 1b. Post-hoc multiple comparison analysis revealed that all pairs of comparisons showed statistically significant differences (*p* <  0.05, Tukey-Kramer test for all pairwise comparisons).

The ICG-R15 test revealed that ICG-R15 was the highest (i.e., the lowest liver function) in the high-dose group (4.3 ± 2.1), followed by the low-dose group (3.3 ± 1.4) and control group (1.1 ± 1.3), as shown in Fig. 1c. Post-hoc multiple comparison analysis revealed that the control group was significantly different from the low-dose group and high-dose group; however, there was no significant difference between the low-dose group and high-dose group.

### DCE-MRI

**Fig. 2:**
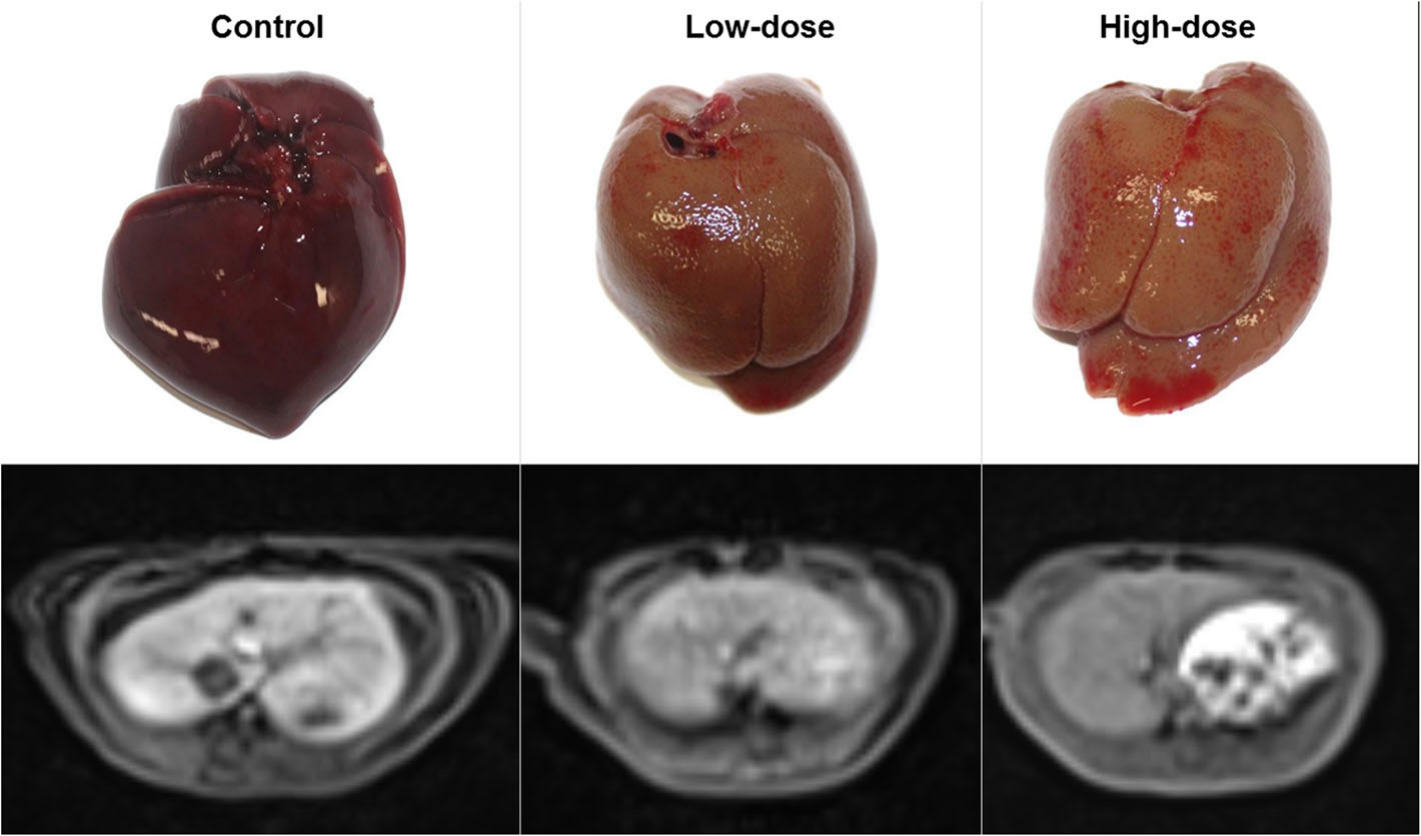
Gross specimen of the liver and gadoxetate-enhanced DCE-MRI at 15 min after contrast injection. In the gross specimens, color of the liver is dark brown in control group, red brown in low-dose group, and light brown with pigmentations in high-dose group. In the MRI, the signal intensity of the liver is highest in control group, followed by low-dose group and high-dose group

In the time-signal intensity curves, the signal intensity of the liver on the gadoxetate-enhanced DCE-MRI was generally highest in the control group, followed by the low-dose group and high-dose group. These findings indicate that the lower enhancement of the liver on the gadoxetate-enhanced MRI reflects the lower liver function and higher liver fibrosis (Fig. 2). Indeed, all the DCE-MRI indices, which were derived from time-signal intensity curves, differed significantly between groups (*p* <  0.001, one-way ANOVA), as presented in Table 1. Post-hoc analysis revealed that the DCE-MRI indices of RLE-3, iAUC-3, iAUC-15, and Emax were different between the control group and low-dose group and between the control group and high-dose group, as shown in Table 1. However, the RLE-15 was different only between the control group and high-dose group.

**Table 1:** Comparison of mean values of the DCE-MRI indices between groups

MRI indices	Control group	Low-dose group	High-dose group	*p* value	Post-hoc analysis^a^
RLE-3	2.84 ± 0.29	1.96 ± 0.23	1.87 ± 0.18	< 0.001	1 vs 2, 1 vs 3
RLE-15	1.93 ± 0.34	1.67 ± 0.05	1.53 ± 0.29	< 0.001	1 vs 3
iAUC-3	6.54 ± 0.51	5.49 ± 0.37	5.20 ± 0.36	< 0.001	1 vs 2, 1 vs 3
iAUC-15	35.37 ± 3.07	28.25 ± 2.09	25.84 ± 2.92	< 0.001	1 vs 2, 1 vs 3
Emax	3.03 ± 0.26	2.28 ± 0.23	2.12 ± 0.20	< 0.001	1 vs 2, 1 vs 3

^a^Pairs with a statistically significant difference in post-hoc analysis. Here, 1 means control group, 2 means low-dose group, and 3 means high-dose group

There was a negative correlation between all MRI indices and the collagen area (%) and between DCE-MRI indices and ICG-R15 (Table 2). The correlation coefficient between DCE-MRI indices and the collagen area was high for iAUC-15, iAUC-3, RLE-3, and Emax (*r* = − 0.81, − 0.78, − 0.80, and − 0.81, respectively) but moderate for RLE-15 (*r* = − 0.51). These results indicated that the all DCE-MRI indices except for RLE-15 can reflect histologic severity of the liver fibrosis.

**Table 2:** Correlation between the DCE-MRI indices and collagen area (%) and between the DCE-MRI indices and ICG-R15

MRI indices	Correlation with collagen area (%)			Correlation with ICG-R15		
	*r*^a^	95% CI	*p* value	*r*^a^	95% CI	*p* value
RLE-3	−0.80	−0.91 to − 0.57	< 0.001	− 0.62	− 0.82 to − 0.27	0.002
RLE-15	−0.51	−0.77 to − 0.12	0.015	−0.56	−0.80 to − 0.18	0.006
iAUC-3	−0.78	−0.90 to − 0.53	< 0.001	− 0.63	− 0.83 to − 0.28	0.002
iAUC-15	−0.81	−0.92 to − 0.58	< 0.001	− 0.65	− 0.84 to − 0.31	0.001
Emax	−0.81	−0.92 to − 0.59	< 0.001	− 0.58	− 0.80 to − 0.21	0.005

^a^Pearson correlation coefficient

The DCE-MRI indices showed moderate negative correlation with liver function based on ICG-R15: the highest for iAUC-15 (*r* = − 0.65), followed by iAUC-3 (*r* = − 0.63), RLE-3 (*r* = − 0.62), Emax (*r* = − 0.58), and RLE-15 (*r* = − 0.56) (Table 2). These results indicated that the iAUC-15 and iAUC-3 are good in assessment of liver function.

The degree of correlation between DCE-MRI indices and liver stiffness measurement by SWE were moderate for iAUC-15 (*r* = − 0.53), iAUC-3 (*r* = − 0.59), RLE-3 (*r* = − 0.58), and Emax (*r* = − 0.57), but negligible for RLE-15 (*r* = − 0.28).

### SWE

Liver stiffness measurement with SWE was successful in all rats without technical failure or unreliable measurement. Liver stiffness were 9.0 ± 2.0 kPa in control group, 12.3 ± 2.3 kPa in low-dose group, and 12.9 ± 2.5 kPa in high-dose group. Liver stiffness differed significantly between groups (*p* <  0.001, one-way ANOVA). Post-hoc analysis showed that the liver stiffness differed between control group and liver fibrosis groups, but did not differ between low-dose group and high-dose group. Liver stiffness measurement results showed a moderate positive correlation with the histologic collagen area (*r* = 0.59, *p* = 0.004) and ICG-R15 (*r =* 0.52, *p* = 0.013).

### Diagnostic accuracy

**Fig. 3:**
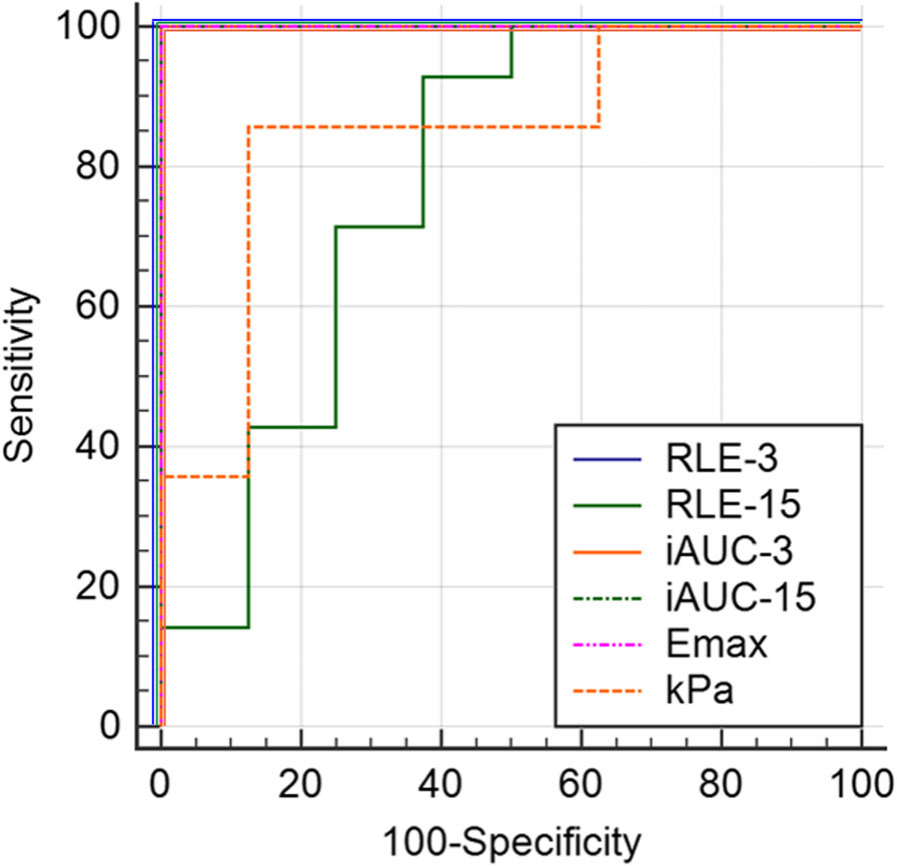
Receiver operating characteristic curves of the DCE-MRI indices (RLE-3, RLE-15, iAUC-3, iAUC-15, Emax) and shear wave elastography index (kPa) for diagnosing liver fibrosis

In the ROC analysis for diagnosing liver fibrosis (i.e., high-dose group and low-dose group), the diagnostic accuracy of RLE-3, iAUC-3, iAUC-15, and Emax was 100% (AUROC 1.000) with complete differentiation between the liver fibrosis groups and control group. On the other hand, the diagnostic value of RLE-15 (AUROC 0.777) and SWE (AUROC 0.848) was lower than that of the other MRI indices (Fig. 3).

## Discussion

In our preclinical study using a liver fibrosis animal model, we demonstrated that gadoxetate-enhanced DCE-MRI is feasible for evaluating histopathologic liver fibrosis and physiologic liver functions in a non-invasive manner. Among the imaging indices of DCE-MRI, the iAUC indices including iAUC-15 and iAUC-3 may be the better indices than RLE indices for evaluating both histopathologic liver fibrosis and physiologic liver functions.

The monitoring of liver function in a non-invasive manner has been emphasized in preclinical trials. However, histopathologic evaluation is still the most common method for the quantification of liver fibrosis, which inevitably requires animal sacrifice. Histopathologic evaluation after sacrificing the animal is limited in that it provides only information at one time point per animal [22]. Liver biopsy might be possible for rats; however, it is also associated with a significant risk of death, hemorrhage, and inflammation/infection. Furthermore, liver biopsy generally acquires only a small piece of tissue, limiting accurate evaluation [8]. Therefore, an imaging approach such as gadoxetate-enhanced DCE-MRI has attracted interest.

The uptake of gadoxetate into hepatocytes occurs via organic anion transporters (OATPs), and the biliary excretion of gadoxetate occurs via multidrug resistance-associated proteins (MRPs) [23]. These receptor-based influx and efflux mechanisms result in unique pharmacokinetic/pharmacodynamic characteristics, contributing to the success of gadoxetate as a hepatocyte-specific MRI contrast agent and allowing the evaluation of liver function. In general, the ICG clearance test and technetium-99 m mebrofenin scintigraphy have been used to estimate liver function because ICG and mebrofenin are substrates for the OATP receptors of hepatocytes and are excreted in the bile through MRP2 [23]. Likewise, since gadoxetate is also a substrate of OATP1B1 and OATP1B3 and is excreted through MRP2, gadoxetate-enhanced MRI can be used to estimate liver function. Liver fibrosis or cirrhosis can reduce OATP and MRP2 levels in the liver parenchyma, leading to reduced enhancement on gadoxetate-enhanced MR images. Therefore, quantitative liver function evaluation is based on the changes in liver parenchymal enhancement on gadoxetate-enhanced DCE-MRI [7]. A recent preclinical study using a cirrhotic rat model also reported reduced liver parenchymal enhancement, which was attributed to slower hepatocyte uptake and rapid elimination due to decreased OATP1 activity and increased MRP2 activity [24].

Liver function estimation using gadoxetate-enhanced DCE-MRI can be categorized into three methods: (1) measurement of liver parenchymal SI in the hepatobiliary phase (i.e., the RLE method), (2) MRI relaxometry such as T1 mapping or T2* mapping, and (3) DCE-MRI with pharmacokinetic modeling [25–28]. In the measurement of liver parenchymal SI, the absolute value of the SI would be different across scans and MRI machines. Therefore, the relative enhancement was calculated by subtracting the SI of the unenhanced images from the SI in the hepatobiliary phase and dividing the difference by the SI of the unenhanced images (i.e., the RLE in our study). Sometimes, the SI is adjusted using internal tissue standards such as the spleen or muscles [25, 26, 29]. The RLE method is very simple to use and does not require sophisticated software. However, its fundamental limitation is that the variability of the enhancement level measured at only one time point is high.

To overcome the limitation of the RLE method, DCE-MRI techniques have been proposed to estimate liver function based on measurements at many time points (i.e., time-intensity curve). This approach allows semi-quantitative analysis or sophisticated pharmacokinetic analysis based on a time-signal intensity curve of hepatic parenchyma and vessels. As DCE-MRI techniques have been greatly improved in the last decade, they are increasingly used for liver function evaluation [28, 30].

In our study, as a translatable index for preclinical trial, we adopted the RLE method to measure RLE-3/RLE-15 and the DCE-MRI method to determine semi-quantitative parameters such as iAUC-3, iAUC-15, and Emax. We did not use the hepatic extraction fraction (HEF), which requires sophisticated modeling and a special software, because it is very difficult to place the ROI on the portal vein and hepatic vein of rats due to the small vessel size. Although the HEF has been widely used in clinical studies, it might not be suitable for small animals such as rats and mice [28, 30]. In our study, the semi-quantitative parameters including iAUC-3, iAUC-15, and Emax generally showed a high correlation between ICG clearance and histopathologic fibrosis. Among the parameters of the RLE method, RLE-3 was good for liver fibrosis assessment, whereas RLE-15 was not. In general, the index calculated from many time points data is more robust than the index of point measurement. In this regard, of the five MRI indices, iAUC-15 and iAUC-3 may be the better indices for estimating liver function and histologic liver fibrosis in preclinical trials.

Liver function evaluation with gadoxetate-enhanced DCE-MRI has several advantages over the traditional ICG clearance test [7]. MRI can evaluate liver anatomy and liver function in localized hepatic abnormalities, which is more clinically relevant than a global assessment [31]. ICG clearance is a method that provides a global assessment of liver function. In addition, gadoxetate-enhanced MRI is non-invasive, whereas the ICG test requires repeated blood sampling, which may result in critical conditions for small animals.

To incorporate gadoxetate-enhanced DCE-MRI in preclinical trials and research for liver function estimation, standardization is a vital prerequisite. The use of several MRI machines and image acquisition techniques may hamper the reproducibility of MRI in estimating liver function [7]. In the same preclinical trial, the same image acquisition and analysis method should be used, i.e., trial-specific standardization [32].

Compared with ultrasonographic SWE, all DCE-MRI indices except RLE-15 showed better correlation with histologic collage area and ICG-R15 and higher diagnostic accuracy of liver fibrosis. However, the large-scale head-to-head comparison research is necessary to further validate the usefulness of DCE-MRI and SWE.

There are limitations in our study. First, the sample size was small, warranting further large-scale experiments. Second, our study used the thioacetamide-induced chronic liver injury animal model, which simulates toxic or drug-induced liver fibrosis. This may limit the generalizability of our study results. In the future researches, it might be necessary to use other animal models for metabolic liver injury, alcoholic liver injury, or cholestatic liver injury.

## Conclusions

In conclusion, in preclinical trials with a liver fibrosis animal model, gadoxetate-enhanced DCE-MRI is a feasible approach for evaluating histopathologic liver fibrosis and physiologic liver functions in a non-invasive manner. Among the five semi-quantitative DCE-MRI indices, the iAUC-15 and iAUC-3 may be the most suitable indices for evaluating both histopathologic liver fibrosis and physiologic liver function. The utilization of gadoxetate-enhanced DCE-MRI in the preclinical trials for new drug development can be helpful for translation of preclinical data into the clinical trials and researches.

## Data Availability

The quantitative data of DCE-MRI, collagen area (%), and ICG-R15 used to support the findings of this study are available from the corresponding author upon request.

## Abbreviations

ANOVA: Analysis of variance
AUROC: Area under the ROC curve
C-0: The initial ICG concentration
C-15: The ICG concentration 15 min after injecting ICG
DCE-MRI: Dynamic contrast-enhanced MRI
Emax: Maximum-enhancement
Gd-EOB-DTPA: Gadolinium ethoxybenzyl diethylenetriaminepentaacetic acid
H&E: Hematoxylin and eosin
iAUC-15: Initial area-under-the-curve until 15-min
iAUC-3: Initial area-under-the-curve until 3-min
ICG: Indocyanine green retention
ICG-R15: Indocyanine green retention at 15-min
MRI: Magnetic resonance imaging
RLE-15: Relative liver enhancement at 15-min
RLE-3: Relative liver enhancement at 3-min
ROC: Receiver-operating-characteristic
ROI: Region of interest
SD: Sprague-Dawley
SI: Signal intensity
